# The concerned significant others of people with gambling problems in a national representative sample in Sweden – a 1 year follow-up study

**DOI:** 10.1186/1471-2458-13-1087

**Published:** 2013-11-21

**Authors:** Jessika Svensson, Ulla Romild, Emma Shepherdson

**Affiliations:** 1 Department of Health Science, Mid Sweden University, Östersund, Sweden; 2 Swedish National Institute of Public Health, Östersund, Sweden

**Keywords:** Problem gambling, Family, Relatives, Concerned significant others, Longitudinal

## Abstract

**Background:**

Research into the impact of problem gambling on close social networks is scarce with the majority of studies only including help-seeking populations. To date only one study has examined concerned significant others (CSOs) from an epidemiological perspective and it did not consider gender. The aim of this study is to examine the health, social support, and financial situations of CSOs in a Swedish representative sample and to examine gender differences.

**Methods:**

A population study was conducted in Sweden in 2008/09 (n = 15,000, response rate 63%). Respondents were defined as CSOs if they reported that someone close to them currently or previously had problems with gambling. The group of CSOs was further examined in a 1-year follow up (weighted response rate 74% from the 8,165 respondents in the original sample). Comparisons were also made between those defined as CSOs only at baseline (47.7%, n = 554) and those defined as CSOs at both time points.

**Results:**

In total, 18.2% of the population were considered CSOs, with no difference between women and men. Male and female CSOs experienced, to a large extent, similar problems including poor mental health, risky alcohol consumption, economic hardship, and arguments with those closest to them. Female CSOs reported less social support than other women and male CSOs had more legal problems and were more afraid of losing their jobs than other men. One year on, several problems remained even if some improvements were found. Both male and female CSOs reported more negative life events in the 1 year follow-up.

**Conclusions:**

Although some relationships are unknown, including between the CSOs and the individuals with gambling problems and the causal relationships between being a CSO and the range of associated problems, the results of this study indicate that gambling problems not only affect the gambling individual and their immediate close family but also the wider social network. A large proportion of the population can be defined as a CSO, half of whom are men. While male and female CSOs share many common problems, there are gender differences which need to be considered in prevention and treatment.

## Background

Problem gambling is regarded as a public health issue [1-3] and is often defined as “gambling behaviour that creates negative consequences for the gambler, others in his or her social network, or for the community” [4]. Previous studies have shown that problem gamblers suffer higher levels of health-related problems than the general population [5-7]. Problem gamblers report lower levels of general and mental health than others, and suffer from higher rates of depression, anxiety and suicide ideation than the general population [8-14].

Concerned significant others (CSOs) is a term used in earlier research [15] and will be employed in this paper to describe people in the surrounding environment of a problem gambler. In this paper, a CSO can be a parent, spouse, child, relative but also a friend or colleague. This definition is coherent with Hodgins et al. [15] while it is broader than the definition of Wenzel et al. [16] who examined only family members (15-16) and Ingle et al [17] who included spouses and boyfriends/girlfriends. Every problem gambler is estimated to have between 7-16 CSOs who could be affected by his or her gambling [18-20]; the range depends on how CSOs are defined. The Australian Productivity Commission [20] reported that almost half of all problem gamblers (49.4%) live in households with an average of two children, while their own survey estimated that there are statistically 0.6 children (under the age of 15 years) living with the average problem gambler. Studies have also examined and confirmed the association between being a problem gambler and having a parent with a problem gambling history [21,22]. However, little attention has been directed to the negative consequences of problem gambling on personal and social relationships [23,24], and the empirical research on consequences for close relatives and friends is surprisingly scarce [25,26]. There is extensive research to suggest that consequences for CSOs range from economic problems to social and health problems [27], however, it has used help-seeking populations or convenient samples to explore the situation of CSOs [17].

Most of the research to date on the impact of problem gambling on CSOs has focused on the female spouses of problem gamblers [16,23,28-30]. Spouses have reported harassment and legal threats by the gambler’s creditors, distress in their children, additional responsibilities arising from the gambler’s absences and neglect of family and physical manifestations such as headaches, sleeping difficulties, depression and anxiety [31-33]. Suicide attempts by spouses and partners of problem gamblers are reported to be three times that of the general population [34,35]. The National Gambling Impact Study [36] estimated the lifetime divorce rates for problem and pathological gamblers were 39.5% and 53.5% respectively; the same rate for non-gamblers was 18.2%.

Research on help-seeking populations has found that spouses of problem gamblers are exposed to higher levels of domestic violence than those with a non-gambling partner. Korman et al. [37] showed that 25.4% of problem gamblers reported that they perpetrated severe intimate partner violence. This result is consistent with findings from a previous study [38], and from earlier research on spouses [32,39] and perpetrators [40]. Children of problem gamblers have been reported to be at risk of experiencing abuse by both the gambler and his or her spouse [41]. Lesieur and Rothschild [42] suggested that children of problem gambling parents are two to three times more likely to be abused than their peers. However, Schluter et al. [43] found no association between being a relative of a problem gambler and being exposed to domestic violence in a longitudinal research study on South Pacific families in New Zealand.

The understanding and treatment of clinical problem gambling is crucial for supporting the concerned individual and his or her family. However, a public health perspective addresses not only the risk of problems for the individual gambler but also the quality of life of the families and communities affected by gambling and acts to prevent the problems at a more structural level [1]. From a public health perspective, efforts targeted at an individual level are often of limited value for society as a whole [44]. We are not able to make more general assumptions from non-representative samples so therefore, knowledge based on epidemiological data is important.

One identified study has explored the epidemiology of problem gambling in the family using a national representative [16]. This Norwegian study showed that problem gambling has a strong impact on the quality of life of CSOs and that 2.0% of the population was a CSO of a problem gambler. Most of the CSOs were women, urban dwellers, divorced, and had unsatisfactory financial situations and unsatisfactory subjective health. The effects of gambling on the CSOs were worsening family financial situations, decreased social relationships, and increased conflicts levels within the family. They also reported greater mental health problems than in the general population, including sleep disorders, depression symptoms, obsessive-compulsive symptoms, alcohol problems, substance abuse and their own problem gambling [16]. Exposure to violence was not included as an outcome in the study. However, the study had a low response rate (36.1%) and in addition, it did not include any separate analyses for men and women.

The proportion of problem gamblers in Sweden seems consistent at just over 2% of the total of the population: 3.2% of the male population and 1.3% of the female population. The highest proportion of problem gambling is found amongst men aged 18-24 years old with close to 1 in every 10 men having a gambling problem. In the oldest age group (65-84 years old) the proportion of problem gambling is higher among women than men [45]. As reported in the first section of this paper; problem gamblers are exposed to various problem regarding health, finances and social relations. Close to 4% of the population (n = 262,500) live in the same household as a problem gambler. Further, close to one third of problem gamblers live in a household that includes children [45].

The hypotheses in this study are based on the findings of Wenzel el al. [16] that CSOs were more likely to be women, were associated with problem gambling, riskier alcohol consumption, and experienced more mental health problems. Additional research questions are included such as social relations, financial situation and important life events from a longitudinal perspective.

### Aim

There is a paucity of evidence regarding the health, social support and financial situations of male and female CSOs of individuals with gambling problems. The aim of this study is to test the hypotheses that women are more likely to be defined as CSOs and that being a CSO is associated with problem gambling, riskier alcohol consumption and increased mental health problems. These hypotheses will be investigated by data from CSOs drawn from a larger Swedish national representative sample including both the baseline study, *Wave I* and the 1-year follow up, *Wave II*. All respondents to the study were asked in both waves if someone close to them, as far as they knew, had or previously had problems with gambling. An additional aim is to answer research questions about 1) if male and female CSOs are more likely than the general population to experience social, financial and health problems, 2) if there is a association between being a CSO and health and life events such as separations, financial problems and legal or work-related problems in the 1-year perspective, and 3) how many CSOs in the baseline study, Wave I, were no longer defined as such one year on in the follow up study, Wave II (ex-CSOs), and are there differences between them and those that still defined as CSOs?

No identified study has discussed gender differences in CSOs based on data from a national representative sample. Therefore this study will perform separate analyses for men and women.

This study will report on the findings to the hypotheses and research questions regarding CSOs from two measurement points with one year between the measurements from a national representative sample in Sweden. Further, it will discuss the implications of the findings.

## Methods

Data was derived from a Swedish longitudinal survey on gambling and health (Swedish Longitudinal Gambling Study - Swelogs). The study used data from the two first measurement points, the baseline study, Wave I and the 1-year follow-up study, Wave II. Both Wave I and II used the same methods of data collection. The primary method was computer-based telephone interviews conducted by Statistics Sweden. Mail surveys and reminders were sent to non-responses.

Wave I used a stratified simple random sample based on gender, age group and estimated risk of problem gambling (being male, low income earner, or living with family members on social welfare) of 15,000 participants. The estimated risk was calculated from register variables according to results from a pilot study conducted in spring 2008.

The frame population consisted of 7,320,367 persons aged between 16 and 84 years in the Swedish Total Population Register. The stratified sample was drawn from the frame population. Calibration weights were used to adjust the results to the frame population.

The total number of respondents (n = 8,165) corresponded to a weighted response rate of 63%. The internal attrition for the question regarding being a CSO was 3.3% (n = 264).

Wave II was conducted from December 2009 to March 2010. The 8,165 individuals who were respondents in Wave I were re-contacted and new calibration weights were calculated. The weighted response rate for the sample in Wave II was 74% (n = 6,021). There was no significant difference between the attrition of CSOs and the general population. In addition, we found no differences in the attrition when comparing male and female CSOs and the general population regarding age, country of birth or family situation. Male CSOs who only participated in Wave I were slightly more likely to be on social welfare than other men while female CSOs who only participated in Wave I were significant more likely to have a lower level of education compared with women in the general population.

A central question in the study is the definition of CSOs. All respondents were asked if someone close to them - as far as they knew - had or previously had problems with gambling. Respondents answering “yes, one” or “yes, several” in Wave I were defined as CSOs and were analysed for research question 1-2 (Table 1). Respondent answering “yes” in both Waves were compared with respondents who only defined as CSOs in Wave I, who were defined as ex-CSOs (Table 2).

**Table 1:** **Odds ratios for factors on health, financial hardship and social support for both Wave I and II**^**a**^

		**PHASE I**				**PHASE II**			
		**OR Crude (95% CI)**		**OR Controlled for age and PGSI (95% CI)**		**OR Crude for CSOs (95% CI)**		**OR for CSOs Controlled for age and PGSI (95% CI)**	
		**Men**	**Women**	**Men**	**Women**	**Men**	**Women**	**Men**	**Women**
Health	Problem gambling (PGSI3+)	3.2 (2.2–4.6)***	0.7 (0.3–1.6)ns	–	-	4.1 (2.6–6.5)***	0.7 (0.2–1.7)ns	-	-
	Good health	1.0 (0.8–1.2)ns	0.9 (0.8–1.1)ns	0.8 (0.7–1.0)ns	0.8 (0.6–0.9)*	1.1 (0.8–1.3)ns	1.0 (0.8–1.2)ns	0.9 (0.7–1.1)ns	0.8 (0.6–1.0)*
	Mental health problem (Kessler 6)	1.7 (1.4–2.0)***	1.8 (1.5–2.2) ***	1.5 (1.2–1.7)***	1.6 (1.4–1.9)***	1.8 (1.5–2.2)***	2.1 (1.7–2.5)***	1.6 (1.3–2.0)***	1.9 (1.5–2.3)***
	Risky alcohol behavior (AUDIT)	2.4 (2.0–2.9)***	1.8 (1.4–2.3)***	2.0 (1.6–2.4)***	1.4 (1.0–1.8)*	2.8 (2.2–3.4)***	1.7 (1.2–2.5)**	2.1 (1.7–2.7)***	1.4 (0.9–2.0)ns
	Sick leave (self-reported)	0.9 (0.6–1.2)ns	1.5 (1.2–1.9)**	1.1 (0.8–1.5)ns	2.0 (1.6–2.5)***	-	-	--	-
Social support and fear of losing employment	Fear of losing employment	1.7 (1.3–2.1)***	1.1 (0.8–1.5)ns	1.6 (1.2–2.1)***	1.0 (0.7–1.4)ns	1.2 (0.9–1.7)ns	0.9 (0.6–1.4)ns	1.2 (0.8–1.7)ns	0.8 (0.6–1.3)ns
	Practical help	1.2 (0.8–1.8)ns	0.6 (0.3–0.9)*	1.1 (0.7–1.6)ns	0.5 (0.3–0.8)**	-	-	-	-
	Someone to share feelings with	0.9 (0.7–1.2)ns	0.7 (0.5–1.0)*	0.9 (0.7–1.2)ns	0.5 (0.4–0.7)***	-	-	-	-
	Violence in last 12 months	3.3 (2.4–4.4)***	7.2 (4.5–11.5)***	2.6 (1.9–3.5)***	5.9 (3.7–9.4)***	-	-	-	-
Financial hardship	Difficult pay bills in last 12 months	1.8 (1.4–2.2)***	1.7 (1.4–2.1)***	1.6 (1.3–2.0)***	1.5 (1.2–1.9)***	1.6 (1.2–2.1)**	2.5 (2.0–3.3)***	1.2 (0.9–1.7)ns	2.3 (1.7–3.0)***
	Living on social welfare	1.9 (1.3–2.8)**	1.8 (1.3–2.6)**	1.5 (1.0–2.2)*	1.7 (1.2–2.5)**	-	-	-	-
Life events in last 12 months	More argument with someone close	-	-	-	-	2.6 (1.8–3.6)***	2.5 (1.8–3.4)***	2.0 (1.4–2.9)***	2.1 (1.5–2.9)***
	Problem at work	-	-	-	-	1.4 (1.0–1.9)*	1.4 (1.0–1.9)*	**1.3 (0.9–1.7)ns**	1.4 (1.0–1.8)*
	Legal problems	-	-	-	-	2.6 (1.8–3.8)***	1.3 (0.8–2.2)ns	2.4 (1.6–3.6)***	1.3 (0.8–2.1)ns
	Divorce or separation	-	-	-	-	1.6 (1.2–2.3)**	2.2 (1.5–3.1)***	1.4 (1.0–1.9)ns	1.9 (1.4–2.8)***
	Worse economy	-	-	-	-	1.3 (1.0–1.8)ns	1.2 (0.9–1.6)ns	1.2 (0.9–1.6)ns	1.2 (0.9–1.6)ns
	Better economy	-	-	-	-	1.2 (0.9–1.7)ns	1.2 (0.9–1.7)ns	1.0 (0.8–1.3)ns	1.1 (0.8–1.5)ns

*p < 0.05, **p < 0.01, ***p < 0.001.

^a^Bold text indicate significant findings.

**Table 2:** **Comparison between Concerned Significant Others in both waves and CSOs in only Wave I (unweighted)**^**a**^

		**Proportions in sample**		**OR**	**Proportions in sample**		**OR**
		**Male non-CSOs (n = 303)**	**Male CSOs (n = 342)**	**(95% CI)**	**Female non-CSOs (n = 253)**	**Female CSOs (n = 329)**	**(95% CI)**
Health	Problem gambling (PGSI 3+)	5.9 (18)	8.5 (29)	1.5 (0.8–2.7)ns	2.4 (6)	4.6 (15)	2.0 (0.7–5.1)ns
	Good health	85.5 (259)	88.9 (304)	1.4 (0.9–2.2)ns	79.0 (199)	76.9 (253)	0.9 (0.6–1.3)ns
	Mental health problems (Kessler 6)	**37.5 (113)**	**47.4 (161)**	1.4 (1.1–2.1)*	**54.6 (136)**	**66.4 (213)**	1.7 (1.2–2.4)**
	Risky alcohol behavior (AUDIT)	35.2 (106)	38.2 (130)	1.1 (0.8–1.6)ns	12.7 (32)	17.5 (57)	1.4 (0.9–2.3)ns
Financial hardship	Difficulties paying bills	12.8 (36)	15.4 (49)	1.2 (0.8–2.0)ns	**21.5 (49)**	**30.5 (93)**	1.6 (1.1–2.4)*
Life events in last 12 months	More arguments with someone close	**10.0 (30)**	**16.7 (57)**	1.8 (1.2–2.9)***	**16.8 (42)**	**29.8 (97)**	2.1 (1.4–3.2)***
	Divorce or separation	11.3 (34)	13.5 (46)	1.2 (0.8–2.0)ns	11.2 (28)	14.8 (48)	1.4 (0.8–2.3)ns
	Problems at work	11.2 (29)	13.8 (42)	1.2 (0.8–2.1)ns	15.2 (32)	21.6 (59)	1.5 (0.9–2.5)ns
	Legal problems	**6.9 (21)**	**12.4 (42)**	1.4 (1.1–3.3)*	7.2 (18)	11.4 (37)	1.6 (0.9–3.0)ns
	Death of someone close	27.2 (82)	29.5 (101)	1.1 (0.8–1.6)ns	**23.1 (58)**	**34 (111)**	1.7 (1.2–2.5)**

Chi-square test and logistic regression^a^.

*p < 0.05, **p < 0.01, ***p < 0.001.

^a^Bold text indicate significant findings.

### Measurements

The study design, the questionnaire and the interview questions in Swelogs were constructed and agreed on by the Swedish National Institute of Public Health with the support of the Swelogs Advisory Board including international experts on gambling. The final questionnaire in Swelogs included questions regarding gambling, gambling problems, health, security, social relations, occupation, economy, living environment, close relationships and help-seeking. Selected socio-demographic factors included age, country of birth (immigration), education, family situation, whether or not respondents lived in a large city, and income. Considerations in the development of the questionnaire included compatibility between Swelogs, the Swedish national public health survey “Health on Equal Terms” (HET) (conducted annually since 2004) and international longitudinal research projects such as the Victorian Gambling Study in Australia and the Leisure, Lifetime and Lifecycle Project in Canada. All the questions in HET have been tested in a pilot study and the construct validity of each individual question was also tested at Statistics Sweden’s measurements laboratory [46]. Empirical validity tests that provide exact results are difficult to carry out in the psychosocial field, which includes many of the questions within the questionnaire. The questions and instruments from HET were as follows:

Self-reported health: General health measured on a five-degree scale is also one of the questions agreed upon in the EU. Respondents were asked ‘How would you assess your general health?’ The response options were as follows: very good, good, fair, bad and very bad. The response alternatives were collapsed into two categories: having good health (containing the answers ‘very good’, ‘good’ and ‘fair’) and having poor health (‘bad’ or ‘very bad’).

Financial hardship and job security: In both waves, the respondents were asked if they have had difficulties paying bills the last 12 months and if they fear losing employment to measure perceived job security.

Physical violence: Whether the respondent was subjected to physical violence during the last 12 months and during his/her lifetime.

Risky alcohol consumption: Three questions from the Alcohol Use Disorder Identification Test (AUDIT) were asked, that created an index with a maximum score of 12. As in HET; the cut-off was eight for men and six for women. AUDIT was developed by the World Health Organization (WHO) with the aim of identifying persons whose alcohol consumption can damage their health. AUDIT has shown good reliability and validity [46,47].

Social support: Social support was measured by the following two questions: 1) Having someone to turn to when you are in need of practical help was measured with the question: “Is there anyone who can help you when you have practical problems or become ill? For example, to give you advice or support, to lend you things, to help with grocery shopping, to do repairs?” 2) “Do you have someone with whom to share your innermost thoughts or feelings?” Answer formats were “yes”, one”, “yes, several” and “no”. The first question is intended to indicate emotional support and the second question indicates instrumental support. The questions originate from the larger measurement instrument (SS-13), developed by Undén and Orth-Gomer [48].

#### Questions and instruments were not taken from HET

Life events: In the follow-up, questions were asked about significant life events during the last year such as including separations, changes in finances, arguments within the family and legal- or work-related problems. The questions on life events were taken from the Victorian Gambling Study [49].

Problem gambling: This study examines different aspects of problem gambling using the Problem Gambling Severity Index (PGSI). PGSI is one of several instruments that can be used to measure problem gambling in population surveys. PGSI was developed largely as a response to criticism about the other more diagnostic instruments such as Diagnostic and Statistical Manual of Psychiatric disorders (DSMIV) and South Oakes Gambling Screen (SOGS). PGSI has become the standard in population based research in many countries [50].

PGSI consists of nine questions: 1. Have you bet more than you could afford to lose? 2. Have you needed to gamble with larger amounts of money to get the same feeling of excitement? 3. When you gambled, did you come back another day to get the money back? 4. Have you borrowed or sold anything to get money to gamble? 5. Have you felt that you might have a problem with gambling? 6. Has gambling caused you any health problems, including stress or anxiety? 7. Regardless of whether you think it was true, have people criticised your betting or told you that you have a gambling problem? 8. Has your gambling caused any financial problems for you or your household? 9. Have you felt guilty about the way you gamble or what happens when you gamble? The maximum possible score is 27. Commonly the PGSI-population is divided into four groups: no problems (0), low risk (1-2), moderate risk (3-7) and problem gambling (8+). In this paper moderate risk and problem gambling was merged into a group called problem gambling (PGSI 3+).

Help-seeking on the behalf of others was measured by the question if the respondents had sought help or information on someone else's behalf for problems with gambling. “Others” here does not necessarily imply a close relationship to the person on whose behalf the information and help-seeking was made.

Questions on exposure to violence, social support, if they ever had lent money to someone for gambling or to pay gambling debts and absence from work because of illness, were only asked in Wave I.

Ethical approval was given by the Examination Board for Ethical Research at the Umeå Regional Ethical Review Board in Sweden.

### Statistical analysis

First, descriptive analyses of the prevalence and socio-demographic distribution of being a CSO in Wave I were presented as proportions together with chi-square tests, for men and women separately (weighted, Table 3) which answered the hypothesis that women wore more likely to be CSOs. The results were further analysed and tested by logistic regression and presented as odds ratios (OR). 95% confidence intervals (CIs) were computed for the ORs. Second, bivariate analyses as well as estimations of a series of logistic regression models were performed to examine how being a CSO affected the probability of having poor health, minimal social support and financial hardship for men and women separately in Wave I (Hypothesis that CSOs are associated with problem gambling, riskier alcohol consumption and mental health problems and research question 1, Table 1). Both crude and adjusted analyses were performed. Age and problem gambling were controlled for as confounders in the adjusted models. Finally, the same series of logistic regression models were performed for the follow-up study Wave II, with new calibration weights.

**Table 3:** Sample description and comparison between CSOs and non-CSOS for men and women

		**Men**		**Women**		**OR (95% CI)**^**a**^	
		**Not CSO% (n)**	**CSO% (n)**	**Not CSO% (n)**	**CSO% (n)**	**Men**	**Women**
Age		***		***		***	
	16–17	3.6 (115)	2.7 (21)	3.1 (103)	3.0 (21)	2.2 (1.3–3.8)**	2.4 (1.4–4.1)**
	18–24	10.6 (335)	17.5 (136)	8.9 (292)	14.4 (100)	4.9 (3.5–6.9***	4.0 (2.8–5.6)***
	25–44	30.0 (946)	45.7 (356)	30.5 (999)	46.4 (322)	4.5 (3.4–6.1)***	3.7 (2.8–5.0)
	45–64	34.5 (1091)	27.0 (210)	34.9 (1141)	26.9 (187)	2.3 (1.7–3.2)***	1.9 (1.4–2.6)***
	65–74	21.2 (671)	7.2 (56)	22.5 (737)	1.6 (64)	1.0	1.0
Education		**		***			
	Low level	28.0 (809)	27.0 (204)	25.2 (756)	17.9 (121)	1.2 (1.0–1.5)ns	0.8 (0.6–1.0)ns
	Mid level	44.4 (1284)	51.1 (386)	42.0 (1258)	52.6 (356)	1.4 (1.2–1.8)***	1.4 (1.2–1.7)**
	High level	27.6 (799)	21.9 (165)	32.8 (984)	29.5 (200)	1.0	1.0
Country of birth		***		ns (0.05)		***	ns
	Born outside Sweden	11.2 (353)	20.1 (156)	15.5 (507)	18.4 (128)	2.0 (1.6–2.4)***	1.2 (1.0–1.5)ns
Family situation		***		***		Controlled for age	
	Single without children	34.1 (1078)	39.7 (309)	31.6 (1034)	32.8 (228)	1.0	1.0
	Single with children	1.7 (65)	2.6 (20)	5.1 (168)	11.1 (77)	1.4 (0.8–2.4)ns	2.6 (1.9–3.5)***
	Married/living with partner without children	40.3 (1273)	27.8 (216)	36.0 (1176)	25.5 (177)	0.9 (0.7–1.1)ns	0.9 (0.7–1.1)
	Married/living together with children	18.8 (741)	29.9 (233)	27.3 (893)	30.6 (213)	1.4 (1.1–1.7)**	1.2 (1.0–1.5)ns
Living on social welfare		**		**			
		3.1 (93)	5.7 (43)	3.7 (115)	6.4 (43)	1.9 (1.3–2.8)**	1.8 (1.3–2.6)**
Have lent money to someone they thought or knew would use it to gamble or pay gambling debts		***		***			
		6.3 (199)	25.4 (195)	1.4 (47)	13.1 (90)***	5.0 (4.1–6.3)***	10.3 (7.2–14.9)***

*p < 0.05, **p < 0.01, ***p < 0.001.

^a^Family situation was controlled for age in the logistic regression.

Regarding research question 3), we only examined respondents who were defined as being a CSO in Wave I. Ex-CSOs in Wave II were compared to those who still were defined as CSOs. Chi-square tests were used for men and women separately and were further described and tested by using ORs from logistics regressions. Age and problem gambling were controlled. Only unweighted data was used as a subsample was analysed (Table 2).

All analyses were made using SPSS version 20.

## Results

In the total sample, 18.2% (n =1,472) defined as a CSO. Men were slightly more likely to be a CSO at 19.5% (n = 778) compared with 17.5% (n = 694) for women (OR 1.2; p < 0.01). This result dismisses the hypothesis that women are more likely to be CSOs. As Table 3 shows, few people aged 65 years old and older or people with a higher level of education were CSOs. Being an immigrant was associated with being a CSO for men only. Further, for men being a CSO was most prevalent among singles with and without children, and married men with children. The last group was only significant after being controlled for age. The family situation for women was less clear, even if single mothers were over represented and married women or women who lived together with a partner without children were under represented. When age and problem gambling was controlled for, only being a single mother was a significant factor.

All CSOs had lent money to someone who they thought or knew would use it to gamble or pay gambling debts. Approximately one third of men who were CSOs had done this compared with 13% of the women who were CSOs. However, the OR was higher for women than for men. The explanation for this is found in the fact that men who were not CSOs also (to a relatively high degree) had lent money to someone for gambling purpose (6.3%). A low proportion of both male and female CSOs had sought help, 8.5% of male CSOs (n = 66) and 10.9% female (n = 75); thus, there was no significant difference between sexes (not shown in table).

### Health among CSOs

The hypotheses that CSOs would be more likely to experience problem gambling, riskier alcohol consumption and mental health problems were partly confirmed. Table 1 shows that in both the crude analyses and after controlling for age and problem gambling, men and women who were CSOs experienced poorer mental health than the general population and had a higher degree of risky alcohol consumption. However, it was only male CSOs who were more likely to be problem gamblers while there was no such association for women. When controlling for age and problem gambling, women reported poorer health than the general population. Women also reported more sick leave days from work – there was no such association for men.

### Social support and fear of losing employment among CSOs

In both the crude and adjusted models, female CSOs were less likely to have someone who could help them with practical issues or someone to talk to and share feelings with than the general population and male CSOs. When controlled for age and problem gambling this association become stronger. There was a greater association between male CSOs and the fear for losing employment in all models in *Wave I*. Even though being a CSO was significantly associated with having been subjected to violence the last 12 months in both models and for men and women, women were exposed to significantly higher levels than men (Table 1).

In both the crude and adjusted models, female CSOs were less likely than the population and male CSOs to have someone who could help them with practical issues if needed, as well as someone to talk and share feelings with. Controlled for age and problem gambling this association became stronger. Female and male CSOs differed in regard to their fear of losing their job. Male CSOs were more associated with a fear of losing their job in all models in Wave I. Even though being a CSO was significantly associated with having been subjected to violence the last 12 months in both models and for both men and women, women were significantly more exposed to violence than men (Table 1).

### Financial situation among CSOs

No gender differences were found regarding financial situations. All CSOs had generally more difficulties than others paying bills (Table 1).

### CSOs – one-year follow up

When analysing the health variables in Wave II, both female and male CSOs still reported significant poorer mental health than the general population. In addition, in the crude analysis both female and male CSOs were still associated with risky alcohol consumption. However, the adjusted model showed that the association became weaker for women in Wave II than in Wave I. Neither female nor male CSOs reported poorer general health than non-CSOs but female CSOs had poorer self-reported health than other women in the models controlling for age and problem gambling. There were no changes in problem gambling: male CSOs remained problem gamblers to a higher degree than both other men and female CSOs (Table 1).

Respondents were asked if they had experienced more arguments with someone close to them in the last 12 months, and if they had experienced a divorce or separation. Compared with ex-CSOs, CSOs generally reported more arguments and separations. In addition, more male CSOs reported that they had encountered legal problems in the last 12 months. However, in Wave II the association between being a CSO and being afraid of losing employment had disappeared. Male CSOs were more likely to have a person close to them die in the last year compared to men in the general population. This was not evident for female CSOs, even though the result was almost significant when controlled for age and problem gambling (Table 1). Both male and female CSOs had a higher probability of having had problems at work during the last 12 months in the crude analysis. Controlled for age and problem gambling, this factor was only predictive for female CSOs. Regarding finances, both female and male CSOs still found it difficult to pay bills in the crude analysis. However, while the association disappeared for men when controlling for age and own problem gambling, it was significant for female CSOs. The finances of male or female CSOs became neither better nor worse even though it was close to significant for both sexes and circumstances (Table 1).

### CSOs at both time points compared with ex-CSOs

The unweighted analyses of Wave II data revealed that 47.4% of the CSOs in Wave I reported that they had no one close to them who had or previously had problems with gambling in Wave II (n = 556). There was no significant difference between sexes (men 47% and women 43.5%). There were no large differences regarding age, even though both men and women aged 45 years old and older were less likely to still be defined as CSOs. Table 2 shows the analysis findings for the CSO sample. We found no differences between CSOs and ex-CSOs regarding problem gambling, self-reported health, alcohol, divorce or further problems at work since compared to 12 months ago for neither sex (Table 2). However, there were other significant differences. Both men and women who were no longer defined as CSOs reported improved mental health and fewer arguments with people close to them compared to 12 months ago. Compared with women who remained CSOs, female ex-CSOs had fewer difficulties paying bills and experienced fewer deaths among those close to them. Male ex-CSOs had fewer legal problems during the past 12 months than men still defined as CSOs in Wave II. Controlling for age and problem gambling did not change any results; except that we found a significant relationship between being a CSO and having experiencing the death of someone close to them during the past 12 months for women (Table 2).

## Discussion

In contrast to the hypothesis that women were more likely than men to be CSOs, this study showed that men are equally likely or even more likely, to be CSOs. Further, men essentially face the same difficulties as female CSOs. However, Swedish statistics from a problem gambler helpline show that women represent the majority of close relatives of problem gamblers seeking help, 80% of annual calls from close relatives are from women [51]. Only a small number (approximately 10%) of the CSOs in this study had sought help which indicates that various services should improve their accessibility and knowledge on issues for CSOs of problem gamblers. Earlier research has shown that the involvement of significant others in treatment is helpful for the problem gambler [52,53], a study of the impact of significant others (e.g. spouses and partners) to 4,411 problem gamblers who were discharge from treatment found that having a significant other was associated with the odds of effective treatment [17].

The hypothesis that CSOs were more likely to be associated with problem gambling, riskier alcohol consumption and mental health problem was partially supported. Prevalence rates from Wave I show that all CSOs experienced mental health related problems and had riskier alcohol consumption than the general population. However, only men who were CSOs were associated with own problem gambling.

As stated, while Wave I showed similarities between male and female CSOs, important gender differences were found. As noted above, men who were CSOs were more likely to be problem gamblers than men in the general population and than women in general including women who were defined as CSOs. Female CSOs reported a worse general health situation in terms of self-reported general health and sick leave days as well as months of absence from work because of illness while male CSOs reported more fear of losing employments. Male CSOs were less absent from work because of illness than other men. Male CSOs also reported more legal problems both at Wave I and II, and male ex-CSOs had fewer legal problems than men who were CSOs at both time points. Female CSOs reported a lack of social support. It is clear from these gender differences that male CSOs, consistent with studies on male problem gamblers, experience more work, debt and legal problems. In contrast, female CSOs, like female problem gamblers, tend to experience problems with relationships and have more physical and mental difficulties [46]. However, in the analyses of CSOs at Wave II, women defined as CSOs at both time points were more likely to have problems at work. This was not found for men who were defined as CSOs at both time points.

Our findings regarding experiences of violence confirmed the results of Brasfield [40], Muelleman et al [39], Korman et al [37] and Bland et al [38]. In contrast, Schluter et al. [43] did not find any association between being a relative of a problem gambler and exposure to domestic violence. However, the authors suggested this could be because of the study design. Both male and female CSOs experienced more violence than the general population. As we do not know the nature of the relationships between the CSOs and the person with gambling problems, we do not know the context of the violence.

Logically, financial hardship, such as in finding it hard to pay the bills or having to receive social welfare, was associated with being a CSO. Both male and female CSOs were more likely to encounter financial hardship at both time points. Women who defined as ex-CSOs in Wave II reported fewer difficulties in paying bills in Wave II compared with women still defined as CSOs. Both male and female CSOs had lent money to others for gambling purposes. This behaviour was more common among men while the OR was higher for women. The explanation for this is found in the fact that other men in the general population, as opposed to women in the general population, (and to a relatively high degree) had also lent money to someone for gambling purpose or to pay gambling debts.

Even though some studies have shown that women who were married to problem gamblers tended to have long marriages [47], earlier research has shown that problem gambling can damage family relations [16]. The relationship between the problem gambler and the CSO in our study is unknown. However, all CSOs had more arguments with someone close to them and were more likely to have been divorced or separated between Wave I and II than men and women in the general population.

Our results are in line with the findings of Wenzel et al. [16], even if our separate analyses for men and women showed that it was only male CSOs that were more likely to be problem gamblers. This and other gender differences in our results suggest that it is important to look at women and men separately. There are no studies to date that examine CSOs from a gender perspective despite the fact that being a CSO is often interpreted as being female and that samples have been dominated by female partners. The use of combined samples of men and women and the analysis of such samples as one entity or purely female samples can lead to gender effects being ignored or missed. The number of female problem gamblers has been increasing in some parts of the world, including Manitoba, Canada and Australia [54,55]. Thus, further research is needed into the male spouses of female gamblers [21,51], same sex spouses and other types of relationships.

Wenzel et al. [16] found a prevalence rate of 2%; however this study found that 18.2% of the population were CSOs. The Norwegian study had a narrower approach asking each respondent two questions (based on the lie-bet instrument) regarding their close relative. In contrast, this study asked a wider and more open question - did the respondent have someone close to them who has, or has previously had, problems with gambling, of which 18% of the sample answered yes to. Further important findings include the long-lasting effects of the associations between being a CSO and mental health, social relations, and financial hardship. In addition, there seems to be opportunities to change when CSOs - for reasons unknown to us – become ex-CSOs. Wenzel et al. [16] did not control their results for problem gambling or age. Our study only found an association between problem gambling and male CSOs, which made it important to consider in analyses. When controlling for problem gambling, we found a tendency for the OR for men to decrease and increase for women.

## Conclusions

The results of this study should be carefully interpreted in light of the study’s limitations. One central limitation of the study is that we do not know the nature of the relationship between the CSO and the person who was reported to have gambling problems. Since we asked the respondents not only if they had someone close who only *has* gambling problems but also if this person had *previously* had gambling problems, it is possible that the period of gambling problems was a long time ago. Of note is that the person close to the respondent who was reported as having problems with gambling is not defined as a problem gambler because it is the respondent’s own perception of gambling problem that is reported. Even if we do not know the nature of the relationships, they are not likely to be only close family as 18% of the population were defined as CSOs and we know that 4% of the population lives in the same household as a problem gambler. However, even if being a problem gambler as defined by the PGSI 3+ is not comparable with having some kind of gambling problem, the difference between 4 and 18% indicates that the CSOs in this paper also include friends and colleagues. And the whole group is more likely to face various problems. The causal relationship between being a CSO and the problems is however, unknown.

In addition, the differences between male and female CSOs may be due to the fact that men in general have “looser” relations with the individuals with gambling problems (as friends, colleagues, and more distant family members) while for female CSOs, this person could possibly be their partner or close family. There is, as presented in the introduction, research performed on relatives of problem gamblers. However this research has been nearly exclusively clinical. What kind of support is needed for friends or colleagues has not been on the agenda, as well as that more epidemiological data on relatives as well as CSOs (as we define CSOs).

The strengths of the study are that it points out areas of interest, that CSOs are a broad and important group and that the associations between various problems are probably not only found in the close environment but also in the wider social network. A further strength is the longitudinal approach that enables to see changes over a one-year period. However, this design was not used in this study to examine the causal relationships between being a CSO and social, financial and health factors, but we now know how these factors developed for the group over a period of one year. A possible way forward in future studies is to examine different groups of CSOs from a gender perspective. CSOs are important, both for reaching and helping people with gambling problems and for their own sake.

## Competing interest

The authors declare that they have no competing interest.

## Authors’ contributions

JS made the analyses and wrote the article. JS and UR have been personally and actively involved in study design, data collection and analyses. ES has been part of the revision and the language editing of the manuscript. All authors read and approved the final manuscript.

## Authors’ information

Jessika Svensson, PhD in Public Health at the Mid Sweden University. She works partly at the Swedish National Institute of Public Health and primarily works on the Swedish Longitudinal Gambling Study. Contact: Jessika Svensson, Swedish National Institute of Public Health, SE 831 40 Östersund.

Ulla Romild, PhD in Statistics, holds a position as Public Health Planning Officer at the Swedish National Institute of Public Health. She is primarily involved with the Swedish Longitudinal Gambling Study, SWELOGS where she is responsible for the data analysis and also bears the scientific responsibility for the project. Ulla also holds a part time position as Statistical Consultant at The Department of Research and Development (RaD), Levanger Hospital, Health Trust Nord-Trøndelag, Norway.

Emma Shepherdson Public Health Planning Officer at the Swedish National Institute of Public Health.

## Pre-publication history

The pre-publication history for this paper can be accessed here:

http://www.biomedcentral.com/1471-2458/13/1087/prepub
